# NIMHD INVESTMENTS IN RESEARCH ON OLDER ADULTS EXPERIENCING HEALTH DISPARITIES: 2018–2022

**DOI:** 10.1093/geroni/igad104.0994

**Published:** 2023-12-21

**Authors:** Aaron Ogletree, Michelle Doose, Yewande Oladeinde

**Affiliations:** National Institutes of Health, Bethesda, MAryland, United States; National Institutes of Health, Bethesda, Maryland, United States; National Institutes of Health, Bethesda, Maryland, United States

## Abstract

The National Institute on Minority Health and Health Disparities (NIMHD) is the nation’s leading institute on the health of populations experiencing health disparities. These populations include individuals with low socioeconomic status (SES), those living in rural areas, sexual and gender minorities (SGM), and racial and ethnic minorities. Despite the need to advance research on these populations, particularly in old age when health disparities may be most salient, there has been no analysis of NIMHD funding on older adults to date. This portfolio analysis synthesized and described NIMHD’s investments in research on older adults using data from fiscal years 2018 through 2022. The Research, Condition, and Disease Categorization system was used to identify funded projects in the area of Aging; manual review confirmed project eligibility. In-depth project characteristics were extracted and topic areas evaluated. Findings demonstrate that NIMHD funded a total of 98 unique research projects focused on older adults. Of these, 47% focused on the etiology of health disparities, 38% on interventions, and 15% on methods and measurement. The most specified population was racial and ethnic minorities (91%), followed by individuals with low SES (21%), rural older adults (11%), and SGM older adults (8%). Projects focused on diagnostic or clinical care (16%), care coordination (6%), caregivers (6%), and shared decision-making (4%) were limited. Findings highlight opportunities for future research to advance health care and reduce health disparities for the growing proportion of older adults from populations experiencing health disparities.

